# The Role of Insulin Signaling in Hippocampal-Related Diseases: A Focus on Alzheimer’s Disease

**DOI:** 10.3390/ijms232214417

**Published:** 2022-11-20

**Authors:** Qi Liu, Zixu Wang, Jing Cao, Yulan Dong, Yaoxing Chen

**Affiliations:** Laboratory of Anatomy of Domestic Animals, College of Veterinary Medicine, China Agricultural University, Beijing 100193, China

**Keywords:** hippocampus, insulin resistance, memory impairment, type 2 diabetes mellitus

## Abstract

Alzheimer’s disease (AD) is a global concern and has become a major public health event affecting human health. Insulin is a metabolic hormone secreted mainly by the peripheral tissue pancreas. In recent years, more and more evidence has proved that insulin regulates various functions of the brain. The hippocampus, one of the earliest brain regions affected by AD, is widely distributed with insulin receptors. Studies have shown that type 2 diabetes mellitus, characterized by insulin resistance, is closely related to AD, which has drawn extensive attention to the relationship between hippocampal insulin signaling and AD. Therefore, we provide an overview of intranasal insulin administration on memory and its underlying mechanism. We also highlight the molecular link between hippocampal insulin resistance and AD and provide a theoretical basis for finding new therapeutic targets for AD in clinical practice.

## 1. Introduction

Since insulin was first demonstrated to have hypoglycemic effects in 1916, followed by the identification of insulin receptors (IRs), a major role of IRs in the regulation of glucose metabolism in peripheral tissues has been established [1,2]. In the past few decades, insulin receptor (IR) function was thought to be restricted to the periphery, and the brain was traditionally considered an insulin-insensitive organ, largely based on the fact that whole-brain glucose uptake is not affected by circulating insulin levels [3]. Over the past two decades, however, studies in the field have identified a unique role for insulin in the brain. There is increasing evidence that insulin enters the brain and regulates central nervous system (CNS) functions such as eating, depression, and cognitive behavior [4,5,6]. The effects on feeding behavior and metabolism appear to be primarily mediated by the hypothalamic actions of insulin, while cognitive function and memory changes are attributed to its actions in the cortex and hippocampus.

The hippocampus is the center of learning and memory, and its dysfunction contributes to neurodegenerative diseases including Alzheimer’s disease (AD) [7,8]. Studies have shown that IRs are widely distributed in the hippocampus [9]. Whether insulin acts on the hippocampus to affect memory has been of interest. Intranasal delivery routes can effectively deliver insulin to CNS targets in a biologically active form. Although the mechanism has not been clarified, many studies in recent years have shown the ameliorative effect of intranasal insulin on memory impairment in animal models and clinical studies, respectively. Currently, type 2 diabetes mellitus (T2DM) is considered to be very prevalent due to the prevalence of obesity and population aging [10]. Notably, studies have shown that people with T2DM were twice as likely to have cognitive dysfunction [11]. Many clinical and animal models have demonstrated a close link between AD and T2DM pathology, and one of the most important links is insulin resistance [12]. In this review, we use insulin and AD as an entry point, summarize the underlying mechanisms by which insulin affects memory, and discuss the potential molecular link between insulin resistance and AD, which may help the future development of novel targets and new treatment options.

## 2. Insulin and Hippocampus: Memory as a Key Link

The hippocampus highly expresses IRs, so changes in insulin signaling in the brain may have significant effects on the hippocampus. Given the crucial role of the hippocampus in memory processing, there has been much interest in whether insulin regulates memory. Here, we elucidated the effects of altered insulin signaling on memory from animal and human studies and summarized the underlying mechanisms.

### 2.1. Evidence in Animal Studies

Changes in brain insulin levels and IR density, as well as reducing the sensitivity of IRs (i.e., insulin resistance), can lead to changes in insulin signaling. The effect of altered insulin signaling on memory function has been discussed in many animal studies. The blood-brain barrier (BBB) limits the ability to deliver drugs and peptides to the brain, and intranasal delivery provides another solution for insulin to enter the brain [13]. A study showed that intranasal insulin can be detected within 5 min in young CD-1 mice and was still present 60 min after injection [14]. In recent years, many studies have shown that intranasal insulin significantly ameliorates memory impairment in animals in various disease models (Table 1). In these disease models, 0.1–2 IU insulin showed different degrees of protective effect. Taken together, these studies suggested that intranasal insulin had a beneficial effect on memory impairment.

The ameliorative effect of intranasal insulin on memory depends on the normal density and function of IRs. Sufficient evidence has proved that the hippocampus IRs are closely related to learning and memory. Animal models showed that the gene expression of IRs in the hippocampus was upregulated after spatial learning [23], and the ameliorative effect of intranasal insulin on memory impairment was also affected by the levels of IRs [14]. Similarly, another study reported that the specific loss of hippocampal IRs resulted in impaired recognition and spatial memory in mice [6]. In addition, the sensitivity of IRs in regulating memory also plays an important role. Many studies have confirmed that hippocampal insulin resistance led to cognitive dysfunction [24,25]. In addition, insulin resistance was characteristic of T2DM, and type 2 diabetic mice or rats were often accompanied with cognitive dysfunction [26,27]. In conclusion, although many mechanisms remain unclear, changes in hippocampal insulin signaling are shown to regulate memory function.

### 2.2. Evidence in Human Studies

Insulin has been widely used as a drug to treat diabetes in clinics since it was discovered. Studies in humans have shown that intranasal insulin can bypass the BBB and reach the CNS within 1 h of administration [28]. The beneficial cognitive effects of insulin delivery to the CNS via the intranasal route have been demonstrated in a series of studies in healthy people [29,30,31,32]. In vivo animal experiments as well as in vitro studies have enabled an understanding of the ameliorative effects of intranasal insulin on cognitive dysfunction in pathological states [15,16,17,18,19,20]. In recent years, there has been increasing interest in the role of brain insulin signaling in the development of AD pathology and the prevention of cognitive impairment with intranasal insulin administration. Several studies have also explored the effect of intranasal insulin on improving memory deficits in patients with AD or MCI (mild cognitive impairment) clinically in humans (Table 2), and these clinical data consistently indicate the positive effects of intranasal insulin on cognitive function in patients. However, these studies still have their limitations. On one hand, to date, intranasal insulin is a novel treatment for patients with AD or MCI that has only been tested in a few clinical trials. On the other hand, the age and sex of the patient; the methods and criteria used to assess cognitive function; and the type, dose, and duration of insulin administration were all factors used to assess the effect of intranasal insulin on memory [33,34]. Notably, the effect of intranasal insulin on cognitive function was also influenced by *apoe4* gene-carrier status. There has been evidence that ApoE ε4 negative individuals are more sensitive to the cognitive consequences of insulin resistance [33]. Patients with Apoe4 (−) showed more consistent cognitive gains compared to patients with Apoe4 (+) [35]. At present, there is sufficient evidence to show that there are few serious adverse effects observed after clinical intranasal insulin administration [35,36,37]. In conclusion, intranasal insulin has emerged as a potential treatment for neurodegenerative diseases, but further studies are needed to determine its effects on cognitive function.

### 2.3. Mechanisms by Which Insulin Affects Memory

Synaptic plasticity in the hippocampus is thought to underlie learning and memory processes [41]. IRs are enriched at hippocampal synapses, where they have been proposed to modulate synaptic plasticity through interactions with the glutamatergic system [24]. AMPA and NMDA receptors are the two most important ionotropic channels gated by glutamate binding. Insulin has a strong effect on glutamate receptor signaling [24]. Studies have shown that insulin-stimulated phosphorylation of GluN2A and GluN2B subunits in the hippocampus enhanced NMDAR-mediated synaptic transmission [42,43]. In addition, insulin exhibits a strong transcriptional regulatory effect on NMDA receptors and may in turn affect synaptic function by altering the composition and kinetic properties of NMDA receptors. A recent study provided additional evidence for a functional interaction between the insulin and glutamate systems [6]. Deletion of IRs specifically downregulated the expression of the GluA1 subunit of AMPA receptors in the hippocampus. Indeed, it was shown most of the AMPA receptors containing the GluA1 subunit are near the postsynaptic membrane in recycling endosomes and can be rapidly recruited under the stimulation of calcium influx mediated by insulin or NMDA receptors. This was a key molecular mechanism for long-term enhancement (LTP), which was important for learning and memory [44,45,46]. Furthermore, insulin activates mTOR and its downstream translational regulators, 4E-BP1 and p70S6K, to stimulate translation of the dendritic spine protein, PSD95, an important postsynaptic compact protein that is responsible for excitatory synaptogenesis and function maintenance [47]. Insulin also modulates the concentration of several neurotransmitters such as acetylcholine and nitric oxide, and controls the release and uptake of GABA and norepinephrine, which in turn affects synaptic function [48,49,50]. In summary, the expression of glutamate receptors at the postsynaptic membrane, the expression of postsynaptic proteins, and the release of neurotransmitters may all influence synaptic function. Glutamate signaling may be a molecular link between brain insulin and hippocampal synaptic function, and these data underscore the critical role of insulin signaling for memory function.

## 3. The Source of Insulin in the Brain

**Figure 1:**
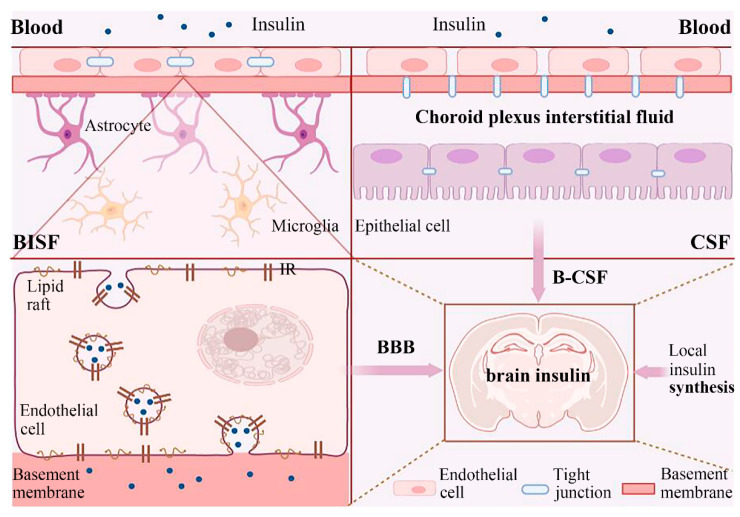
Schematic diagram showing the possible sources of brain insulin. First, the BBB is composed of a capillary basement membrane, pericytes, astrocytes, and specialized capillary endothelial cells that are interconnected with tight junctions. Peripheral insulin can cross the BBB intact through IR-specific vesicle-mediated transport in endothelial cells. Second, the B-CSF barrier has fenestrated capillaries in the choroid plexus that lack tight junctions and allow para- and trans-cellular transport across the endothelium. The B-CSF barrier is another possible route for insulin to enter the CNS. Third, there is some limited evidence suggesting the possibility of de novo insulin synthesis in the brain. BBB: blood-brain barrier, BISF: brain interstitial fluid, CSF: cerebrospinal fluid; IR: insulin receptor.

In recent years, the idea that normal brain function is not insulin-independent has also been revisited. As mentioned above, it has been confirmed in many studies that hippocampal function is affected by insulin. However, there is no doubt that peripheral insulin is produced by the pancreas, so where does insulin come from in the brain? (Figure 1).

### 3.1. External Insulin Reaches the Brain

There was evidence that most IR isoforms in the human and mouse brain were predominantly localized in microvessels [51]. At present, peripheral insulin enters the brain through the BBB and the blood-cerebrospinal fluid (B-CSF) barrier, which are the two most concerning pathways. There is enough evidence that insulin can pass through the BBB into the brain [52]. As a special protective structure, the BBB is composed of a capillary basement membrane, pericytes, astrocytes, and specialized capillary endothelial cells that are interconnected with tight junctions [53]. A new study showed that pancreas-produced insulin interacted primarily with the IR on the luminal side of the brain vasculature [51]. IRs expressed on BBB endothelial cells play a major role in the transport of insulin to the CNS [54,55]. Recent studies have added to this view: in addition to IRs, endothelial cell-mediated insulin transport also requires lipid raft function [55]. Insulin crosses the BBB intact through IR-specific vesicle-mediated transport in endothelial cells. In addition, IRs in astrocytes also mediate insulin transport [56]. It should also not be overlooked that in vivo studies have shown that insulin across the BBB can occur independently of insulin [54], and a similar finding was obtained in another in vitro experiment [57], suggesting that the IRs may not be the only protein-mediated insulin transport in the BBB. These findings greatly increased our understanding of the pathways involved in brain insulin transport. Notably, various other events such as obesity, diabetes, and LPS-induced inflammation alter the permeability of the BBB to insulin, which may lead to changes in insulin signaling and related functions in the brain [58,59,60].

The B-CSF barrier is another possible route for insulin to enter the CNS. The B-CSF barrier has fenestrated capillaries in the choroid plexus that lack tight junctions and allow para- and trans-cellular transport across the endothelium [61]. Earlier findings supported the hypothesis that the choroid plexus has a high density of IRs and suggested that the choroid plexus may be the site of brain insulin transport to the CSF [62]. However, evidence for direct insulin receptor-mediated insulin transport across the choroid plexus is still lacking.

### 3.2. Local Insulin Synthesis in the Brain

The question of whether the CNS secretes insulin has been debated for a long time. Although the evidence was insufficient, previous studies have indicated that partial insulin may also be secreted by the CNS. For example, in the study of Dorn et al., radioimmunoassay analysis revealed much higher concentrations of insulin and C-peptide in the human brain than in the blood, with the highest in the hypothalamus, and immunostaining was mainly restricted to the cell soma and proximal dendrites. They observed immune response products to the two peptides in most nerve cells in all regions of the brain examined [63]. Schechter et al. further proved the presence of insulin in the CNS via rabbit neurons isolated in vitro and indicated that the neurons may be one of the synthesis sites of insulin in the brain [64]. These early studies supported that insulin was at least partly produced in the CNS. However, there are also studies showing that the brain produces little or no insulin [65]. This question has been controversial for many years. It was reported that *Ins2* mRNA was strongly expressed in GABAergic glial cells in the rat cerebral cortex [66]. Nemoto et al. reported that synthesized insulin was secreted from rat hippocampal and cortical neurons’ dense-core vesicles [67]. Moreover, recent studies have reported that astrocytes isolated from the cerebral cortex of rat embryos express *Ins2* mRNA and secrete insulin, which confers strong protection against AβO synaptic toxicity [68,69]. Notably, Aβ, a molecule characteristic of AD, has been reported to reduce insulin synthesis and secretion in cultured neurons and astrocytes and may cause impaired insulin signaling, which also provided new insights into the link between insulin signaling in the brain and AD [67,69]. These studies provide some evidence for the local production of insulin in the brain, and the possibility of the brain synthesizing insulin.

## 4. Insulin Signaling and Hippocampal Disease: AD Is a Key Point

AD is the most common form of dementia, and its most important feature is the persistent and progressive impairment of cognitive function, especially the severe decline in memory. Some human clinical data suggested that people with T2DM, which was characterized by insulin resistance, had a significantly increased risk of developing AD. In recent years, several animal studies have explored the mechanistic effects of insulin resistance on AD. Here, we summarized the molecular link between hippocampal insulin resistance and AD.

### 4.1. The Role of the Hippocampus in AD

**Figure 2:**
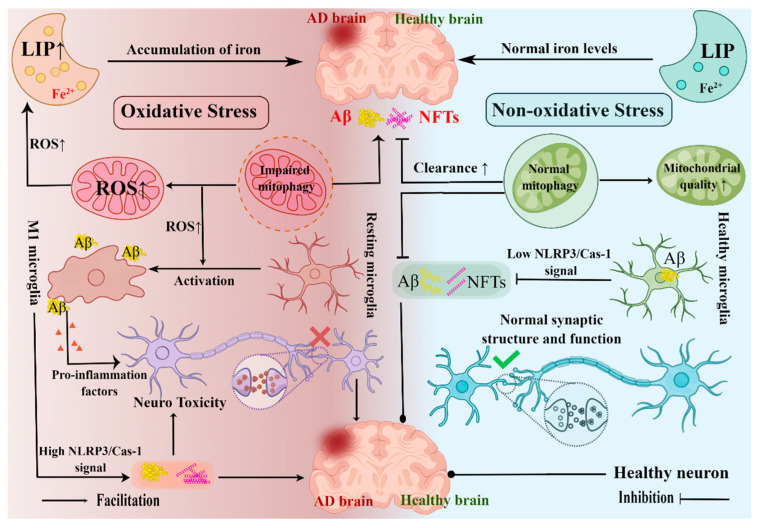
Association between hippocampal pathology and AD disease. AD is characterized by the accumulation of Aβ and hyperphosphorylation of tau protein which lead to neuronal degeneration and changes in synaptic structure and function, leading to neurotoxicity. The damage of mitophagy can lead to the reduction in mitochondrial quality and abnormal mitochondrial function. On the one hand, it promotes the progression of AD pathology. On the other hand, the abnormal mitochondrial function also leads to increased ROS release, which may further lead to hippocampal iron accumulation and neuroinflammation, and then lead to Aβ accumulation and hyperphosphorylation, which may eventually lead to neurotoxicity and AD. AD: Alzheimer’s disease, LIP: Labile iron pool, Aβ: Amyloid beta, NFTs: Neurofibrillary tangles, √: Protective effect, ×: Damaging effect (Drawn by Figdraw).

AD is usually associated with the extracellular deposition of the Aβ peptide and accumulation of hyperphosphorylated tau in neurons. Neuronal degeneration and synaptic changes caused by these pathologies are considered to constitute the main neurobiological basis of cognitive dysfunction in AD [70]. The hippocampus is one of the earliest brain regions affected by AD and reduced hippocampal volume and elevated rates of atrophy have been found in patients with early AD in many structural and functional imaging studies [7,8,71]. Therefore, alterations in hippocampal structure and function may be good candidates for predicting AD development. Here, we summarized the association of hippocampal pathology with the development of AD (primarily Aβ accumulation and tau hyperphosphorylation) (Figure 2).

#### 4.1.1. Hippocampal Neuroinflammation and AD

Neuroinflammation due to microglial activation is thought to play a key role in the ongoing neurodegeneration of AD. Activated microglia secrete a variety of proinflammatory cytokines and toxic products, leading to neuronal dysfunction and apoptosis. The transcription factor NF-κB is known to be a master regulator of inflammatory responses. Studies have shown that activation of NF-κB promoted amyloid precursor protein (APP) cleavage and Aβ production by enhancing BACE1 expression [72]. In AD, reactive microglia adjacent to Aβ plaques have been repeatedly observed in the hippocampus in both clinical data and animal experiments [73,74,75]. Not only that, but the latest research also suggested that microglia carrying being swallowed Aβ would be disseminated to other health areas of the brain, causing the formation of new Aβ [76], and Aβ deposition would continue to cause chronic activation of microglia, leading to excessive production of cytokines and chemokines, thereby deepening the microglia activation and inflammatory response. In addition to affecting Aβ production [77], studies have shown that Aβ activated the NLRP3 inflammasome in microglia to promote tau pathology and neurodegeneration [78,79]. Of note, previous in vivo and in vitro experiments have consistently shown that microglial activation drove the spread of tau tangles [78,80]. A recent study also demonstrated, for the first time from the brains of living patients, that the diffusion path of tau depends on microglial activation [81]. In conclusion, neuroinflammation is an indispensable and a key link in the upstream pathogenesis of AD. Microglia activation is not only a symptom of inflammation, but also very likely to have some association with Aβ pathology and tau pathology, and is a key role in promoting the progression of AD.

#### 4.1.2. Hippocampal Ferroptosis and AD

In recent years, the role of ferroptosis in neurodegenerative diseases has received much attention. Iron accumulation has been observed in the brains of AD patients and AD transgenic mouse models, with excess iron accumulation in insoluble Aβ plaques and neurofibrillary tangles [82,83]. Sufficient evidence has shown a clear link between age-related elevated iron load and AD symptoms [84]. Downregulation of Ferroportin (FPN), the only known iron exporter, may be a key link between iron accumulation and AD [85,86]. Recent studies have shown decreased hippocampal FPN expression and abnormal iron deposition in the brains of AD mouse models and AD patients [84], and that increased brain iron levels may accelerate Aβ formation [87]. Similarly, the administration of specific inhibitors of ferroptosis effectively reduced neuronal death and memory impairment induced by Aβ aggregation in vitro and in vivo [84]. GPX4 is also a central regulator of ferroptosis. It has been reported that the knockdown of GPX4 in mice directly leads to age-dependent neurodegenerative changes and significant neuronal loss [88]. Iron accumulation occurs not only in neurons but also in microglia. On the one hand, iron accumulation in microglia can reduce the phagocytic ability of microglia to Aβ, leading to excessive deposition of Aβ [89,90]. On the other hand, iron accumulation can drive microglia to polarize into the proinflammatory M1 type, thereby inducing neuroinflammation [91]. In general, ferroptosis is a novel form of cell death characterized by intracellular iron overload. Excessive iron accumulation aggravates Aβ accumulation and tau hyperphosphorylation, which provides new insights into the molecular pathophysiology of AD.

#### 4.1.3. Hippocampal Mitophagy and AD

Mitophagy is a form of cellular autophagy that selectively removes defective mitochondria. Corresponding with the age-related increase in AD incidence, there is also an age-dependent accumulation of dysfunctional mitochondria and impaired mitophagy [92]. In biopsies from human AD cases and transgenic animal models of AD, electron microscopic studies have identified the accumulation of damaged mitochondria, such as the appearance of swelling with sclerosis and distortion [93], while basal levels of mitophagy in the hippocampus of postmortem AD patients are 30–50% lower than normal [94]. These studies indicated that mitophagy was dysfunctional in the hippocampus of AD patients [95]. The mechanism of hippocampal mitophagy in AD remains largely unexplored. A recent study found that induction of mitophagy improved AD pathology and reversed memory impairment in transgenic nematodes, IPSC-derived neurons, and mouse models of AD [94]. In APP/PS1 mouse model, mitophagy reduced insoluble Aβ1-42 and Aβ1-40, and inhibited neuroinflammation and cognitive impairment through phagocytosis of Aβ plaques by microglia, suggesting that abnormal mitophagy may be one of the causes of AD. However, other studies have shown that Aβ peptide accumulation in the hippocampus of APP/PS1 mice decreased hippocampal mitochondrial mass and increased mitophagy [96]. A previous in vitro study had consistent results. The accumulation of mAPP and Aβ led to abnormal mitophagy function in hippocampal neurons [97]. These data suggested that abnormal mitophagy may be the initiator of Aβ aggregation and tau hyperphosphorylation, which can further aggravate mitochondrial dysfunction, thus forming a vicious circle in AD pathology.

#### 4.1.4. Hippocampal Oxidative Stress and AD

Oxidative stress, a severe imbalance between the production of reactive oxygen species (ROS) and reactive nitrogen species (RNS) and antioxidant defenses, has been shown to promote the pathological progression of AD in a wide range of studies. In a recent study, the results of single-cell whole-genome sequencing data indicated higher than normal levels of single nucleotide changes associated with oxidative stress and associated DNA damage in the hippocampus and cortex of AD patients [98]. GSH, an enzyme that fights oxidative stress, was significantly depleted in the hippocampal region of patients with MCI and AD compared with healthy elderly subjects [99]. In neurons, accumulated ROS can oxidize polyunsaturated neuronal lipid products to produce active lipid byproducts, such as 4-hydroxy-2, 3-nonenal (HNE), malondialdehyde, and F2-isoprostane or glycosylated proteins to produce advanced glycosylation end products (AGEs). HNE and AGES can also overaccelerate Aβ production and tau phosphorylation. Moreover, recent studies have shown that ROS caused the overexpression of β-site APP cleavage enzyme 1 (BACE1) and increased Aβ production [100], which in turn exacerbated mitochondrial dysfunction and ROS production [101], leading to a vicious cycle. Of note, oxidative stress appears to be at the intersection of many pathological changes, such as neuroinflammation, ferroptosis, and mitochondrial dysfunction.

### 4.2. T2DM and AD

T2DM is a chronic endocrine disease that affects approximately 6% of the global population. The occurrence of T2DM can cause many complications in the body. Currently, the most observed neurological effects of T2DM are impaired learning and memory. Many studies have shown that humans with T2DM exhibited cognitive deficits, characterized by smaller hippocampal size and hippocampal atrophy, and poor memory in T2DM patients [102,103,104]. Similar to these results, in animal studies, T2DM mice/rats performed poorly in many behavioral tests, such as the delayed alternation T-maze task [26], the Y-maze test, the Morris maze water test [105,106], the nest building test, and the novel object recognition test. This evidence supported a strong relationship between T2DM and cognitive function. In addition, clinical and epidemiological studies have demonstrated that the risk of developing AD is twice as high in patients with T2DM compared to those without diabetes [107,108].

The central feature of T2DM is insulin resistance [109]. Subsequent studies have shown that insulin resistance caused hippocampal neuroplasticity deficits [110], leading to decreased performance on hippocampal-dependent learning and memory tasks [111]. Sufficient evidence has been obtained to demonstrate the development of hippocampal insulin resistance in AD patients or AD animal models [112,113]. Collectively, these studies supported the hypothesis that hippocampal insulin resistance was a common pathological feature of T2DM and AD, with some studies also calling AD “type 3 diabetes”.

### 4.3. Molecular Link between Hippocampal Insulin Resistance and AD

**Figure 3:**
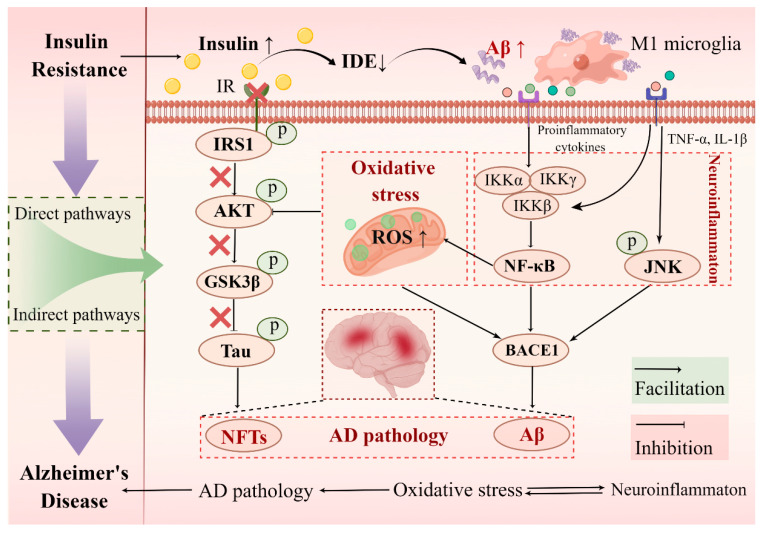
A molecular link between insulin resistance and AD. Insulin resistance contributes to the development of AD pathology through both direct and indirect pathways. Brain insulin resistance reduces IDE levels and insulin signaling directly induces Aβ deposition and tau hyperphosphorylation. In addition, insulin resistance-induced neuroinflammation and oxidative stress are also involved in the regulation of AD pathological progression. IR: insulin receptor, IDE: insulin-degrading enzyme, Aβ: amyloid beta, NFTs: neurofibrillary tangles, ×: inhibiting effect (Drawn by Figdraw).

Hippocampal insulin resistance is characterized by the insensitivity of hippocampal IRs and decreased phosphorylation of insulin downstream signaling molecules. Studies have shown a strong association between hippocampal insulin resistance and AD pathology, including Aβ aggregation and tau hyperphosphorylation [25,114]. Here, we summarized recent findings on the possible mechanisms by which hippocampal insulin resistance induced AD pathology (Figure 3).

#### 4.3.1. Direct Pathways of Hippocampal Insulin Resistance Induced AD Pathology: Aβ Aggregation and Tau Hyperphosphorylation

Aβ peptides are produced by the hydrolysis of APP. The accumulation of Aβ proteins into plaques between cells is considered to be a typical pathological feature of AD [115]. Insulin-degrading enzyme (IDE) is a widely expressed zinc-dependent metalloproteinase that contributes to the proteolytic inactivation of insulin [116]. It is worth noting that Aβ protein is also the substrate of IDE. Studies have shown that IDE plays a crucial role in the clearance of Aβ in AD [117]. Thus, IDE is also considered a link between insulin resistance and AD. In mice, increased γ-secretase activity and decreased IDE activity due to insulin resistance or hyperinsulinemia have been shown to lead to increased Aβ in the brain [118]. In addition, hyperphosphorylation of tau protein is another pathology of AD. Sufficient evidence has shown that tau phosphorylation is regulated by GSK-3β, which is regulated by insulin signaling. Studies have shown that when insulin resistance occurred in the hippocampus, the activity of PI3K/AKT, the main signaling molecule of the insulin signaling pathway, was decreased, which promoted the activation of GSK3β and phosphorylation of tau and promoted the pathological progression of AD [119].

#### 4.3.2. Indirect Pathways of Hippocampal Insulin Resistance Induced AD Pathology: Neuroinflammation and Oxidative Stress

It has been shown that hippocampal insulin resistance led to microglia activation [120], and activated microglia released proinflammatory-related factors (IL6, TNF-a, IL-1β) and induced neuroinflammation. Current studies have shown that these proinflammatory factors can promote Aβ accumulation through three pathways. Firstly, the increase in proinflammatory factors inhibited the phagocytosis of Aβ protein by microglia and then induced the accumulation of Aβ. Second, TNF-α and IL-1β are potent stimulators of γ-secretase, leading to increased Aβ production through pathways involving the c-Jun N-terminal kinase (JNK)-dependent MAPK pathway [121]. Third, activation of the NF-κB pathway has been shown to induce ROS production and accumulation [122]. In addition, ROS may be directly affected by insulin resistance [123]. On the one hand, the increase in ROS induces the increase in BACE1 activity, thereby causing the accumulation of Aβ [124], on the other hand, it induces the generation of oxidative stress. Studies have shown that oxidative stress inactivates the AKT pathway [125], followed by increased cerebral insulin resistance, activation of GSK3β, and phosphorylation of tau. Overall, oxidative stress and neuroinflammation appear to be an important link between hippocampal insulin resistance and AD development.

## 5. Conclusions

It is now widely accepted that insulin in the brain plays an important role in regulating many functions of the CNS. IRs are highly expressed in many brain regions, including the hippocampus. Although mechanistic studies have been insufficiently conducted, adequate animal studies have demonstrated a significant improvement in memory impairment with insulin; however, this improvement has not been as evident in clinical studies. Some studies have shown that clinical trial delivery devices affected the effectiveness of insulin delivery in the CNS, which may be one of the possible reasons for the deviation of results between clinical trials and animal studies [13]. Previous studies have shown that the interaction between insulin and glutamatergic receptors can change hippocampal synaptic plasticity, which may be one of the key mechanisms by which insulin improves memory. As a common link between T2DM and AD, in recent years, insulin resistance has been shown to contribute directly or indirectly to the progression of AD. To date, there is no clinical treatment for AD associated with T2DM. Comparative studies that identify the various pathways involved in insulin signaling may help illustrate the relationship between AD and T2DM or their relative treatment, which may prove potential future research areas.

## Figures and Tables

**Table 1:** Evidence in animal studies.

Dose of Insulin	Time of Intranasal Administration	Animal Models	Memory Detection Method	Effects on Memory	References
Low level (0.0715 IU)	once a day, 5 days a week, 12 weeks	18-month-old male F344 rats	Morris water maze test	No obvious effects	[15]
Low level (0.24 IU)	once a day, 4 consecutive weeks	male C57BL6 mice	Radial arm water maze test	Improvement	[16]
Low level (0.1 IU and 0.5 IU)	once a day, 4 consecutive weeks	kainic acid-induced chronic epileptic mice	Morris water maze test	Improvement	[17]
Low level (0.5 IU)	once a day, 7 consecutive days	Wistar with methamphetamine for 10 days	Y-maze test, Novel object recognition test	Improvement	[18]
High level (1 IU)	twice a day, 14 consecutive days	C57BL/6J mice treated with an I.C.V. injection of STZ	Morris water maze test	Improvement	[19]
High level (1.75 IU)	once a day, 3 consecutive days	3xTg-AD mice anesthetized with propofol/sevoflurane for 3 h	Morris water maze test, novel object recognition test	Improvement	[20]
High level (2 IU)	once a day, 14 consecutive days	rats were injected with STZ (3 mg/kg, ICV) bilaterally twice, on days 1 and 3	Morris water maze test	Improvement	[21]
High level (2 IU)	once a day, 6consecutive weeks	Wistar rats were injected with 6-OHDA (12 μg/4 μL) into the unilateral medial forebrain bundle	T-maze rewarded alternation test	Improvement	[22]

**Table 2:** Evidence in human studies.

Objective (MCI or Mild to Moderate AD)	Dose and Duration of Intranasal Insulin Administration	Memory Detection Method	Effects on Memory	References
289 adults	40 IU/day, 12 months	adas-cog-12 score	No benefits	[13]
60 adults	40 IU/day, 21 days	verbal working memory, visuospatial working memory	Improvement	[38]
104 adults	40 IU/day, 4 months	delayed story recall, the dementia severity rating scale	Improvement	[33]
49 adults	20 IU/day, 12 months	Alzheimer’s disease assessment scale-cognition, Alzheimer’s disease cooperative study-activities of daily living scale, a memory composite	Improvement	[39]
36 adults	40 IU/day, 4 months	global cognition (Alzheimer’s disease assessment scale-cognition)	Improvement	[40]
